# Dietary nutrients’ intake and sleep quality in peritoneal dialysis patients

**DOI:** 10.5935/1984-0063.20200102

**Published:** 2021

**Authors:** Hamed Azarnoush, Mojgan Mortazavi, Mohammad Hossein Rouhani, Shiva Seirafian, Abdolamir Atapour, Mohsen Hosseini, Arash Toghyani

**Affiliations:** 1 Isfahan University of Medical Sciences, Internal Medicine Department, Medical School - Isfahan - Iran (the Islamic Republic of) - Iran.; 2 Isfahan University of Medical Sciences, Isfahan Kidney Diseases Research Center, Alzahra Hospital - Isfahan - Iran (the Islamic Republic of) - Iran.; 3 Isfahan University of Medical Sciences, Food Security Center and Department of Community Nutrition - Isfahan - Iran (the Islamic Republic of) - Iran.; 4 Isfahan University of Medical Sciences, Isfahan Kidney Diseases Research Center, Khorshid Hospital - Isfahan - Iran (the Islamic Republic of) - Iran.; 5 Isfahan University of Medical Sciences, Department of Biostatistics and Epidemiology, School of Public Health - Isfahan - Iran (the Islamic Republic of) - Iran.

**Keywords:** Peritoneal Dialysis, Sleep Quality, Nutrients

## Abstract

Sleep disturbances are common in dialysis patients. However, there is a lack of information on nutritional determinants of sleep disorders in dialysis patients. The objective of the current study was to investigate the association between nutrients’ intake and sleep quality in peritoneal dialysis patients. The cross-sectional study was done on 114 peritoneal dialysis patients referred to Alzahra and Khorshid hospitals, Isfahan, Iran. Information on sleep quality and dietary intakes were collected using Pittsburgh sleep quality index and 168-item food frequency questionnaire respectively. Anthropometric measurements were done by a trained dietitian based on standard protocols. Socio-demographic and clinical data were obtained through a structured questionnaire. The binary logistic regression model was used to detect the association between nutrients’ intake and sleep quality. Our results indicated that there was not any significant difference in basic (socio-demographic and clinical) characteristics between peritoneal dialysis patients with good and poor sleep quality (p>0.05). The results of logistic regression indicated a positive significant association between dietary intake of carbohydrate (OR:3;95% CI:1.32-6.81; p<0.05), fat (OR:3;95% CI:1.32-6.81; p<0.05), and fiber (OR:2.53;95% CI: 1.12-5.67; p<0.05) with poor sleep quality in crude and adjusted models (p<0.05). However, there was not any significant association between dietary intake of protein and poor sleep quality (p>0.05). The results of the present study indicated that dietary intake of nutrients affect sleep quality in dialysis patients. These results help healthcare professionals in making nutritional interventions to improve sleep quality in dialysis patients.

## INTRODUCTION

End-stage renal disease (ESRD) is a major health problem with an increasing prevalence all around the world. Sleep disorders including restlessness leg syndrome, excessive daytime sleepiness, sleep apnea syndrome, and insomnia are frequent in ESRD patients undergoing dialysis^1-3^. The prevalence of sleep disorders in dialysis patients ranged from 40% to 80%^4-6^. Previous studies revealed that sleep disorders are associated with poor health related quality of life and increased mortality in the dialysis patients^7,8^. Thus, it is important to explore risk factors for sleep disorders to enhance dialysis patients’ outcome.

There is no consensus on the etiology of sleep disorders in dialysis patients; although, it is known that various risk factors like psychological disorders, dialysis duration, age, inflammation, and malnutrition are among important determinants of sleep disturbances in ESRD patients undergoing dialysis^1,4^. Few studies have suggested that there is a link between nutritional status of dialysis patients and sleep quality. The results of a study by Li et al. (2012)^10^ on continuous ambulatory peritoneal dialysis patients showed that poor sleep quality is associated with malnutrition and calcium phosphate product^9^. It has also been suggested that poor appetite affect sleep quality in hemodialysis patients^10^. Although, to the best of our knowledge, no previous study addressed the association between the consumption of specific foods and nutrients with sleep quality in dialysis patients.

Since previous studies on the general population have indicated that nutritional factors affect sleep quality, we decided to investigate the association between nutrients’ intake and sleep quality in Iranian patients on continuous ambulatory peritoneal dialysis (CAPD). The results of the study can help healthcare professionals to improve associated quality of life and mortality in dialysis patients through expanding their knowledge on nutritional predictors of sleep quality in dialysis patients.

## MATERIAL AND METHODS

### Study design and patients

The cross-sectional observational study was done on peritoneal dialysis patients referred to two referral dialysis centers (Khorshid and Alzahra hospitals) affiliated to Isfahan University of Medical Sciences (IUMS), Isfahan, Iran. All adult ESRD patients (≥18 years old) who underwent peritoneal dialysis for more than 3 months with the ability to complete questionnaires were included in the study. The study protocol was approved by research ethics committee of IUMS (research project number: 397563) and written informed consent form was obtained from all patients.

### Data collection

A variety of clinical and socio-demographic data comprising age (years), sex (male and female), marital status (single and married), educational status (0-5, 5-12 and >12 years), smoking (yes), body mass index (kg/m^2^), causes of ESRD (diabetes, hypertension and others), and dialysis duration (months) were primarily collected through a structured interview. The Persian validated version of Pittsburgh sleep quality index (PSQI) was applied to assess sleep quality in peritoneal dialysis patients^11^. The self-administered questionnaire consisted of 19 questions and seven component scores including, sleep quality, sleep latency, sleep duration, habitual sleep efficiency, sleep disturbances, using sleeping medications, and daytime dysfunction. The final score ranges from 0 to 21 and higher scores representing poor sleep quality. In this study, patients with a score of ≤5 was considered as good sleepers and those with a score of >5 were classified as poor sleepers.

A 168-item food frequency questionnaire (FFQ) was used to assess dietary intake of peritoneal dialysis patients^12^. Patients were asked to detect their consumption frequency of each food item on a daily, weekly and monthly basis during the preceding year. Daily dietary intake were assessed using nutritionist software IV.

Anthropometric assessments were done by a trained dietitian. Weight was measured to the nearest 0.5 kilogram (kg) and height was measured to the nearest 0.1 centimeter (cm) according to standard protocols. Body mass index (BMI) was calculated as weight in kg divided by height in meters squared (kg/m^2^).

### Statistical analysis

Data for continuous and categorical variables are reported as mean and standard deviation (SD) or percentages across categories of sleep quality, respectively. Differences between groups were examined using independent samples t-test for continuous variables or Mann-Whiney U nonparametric test and chi-square test for categorical variables. Nutrients were adjusted for age (y), sex, and total energy intake (kcal/d) and compared between sleep quality groups by analysis of covariance (ANCOVA). Total energy intake was adjusted for age and sex. Odds ratios (ORs) and 95% confidence intervals (CIs) for the association of macronutrients intake and being in the poor quality sleep group were estimated through binary logistic regression in crude and multivariable-adjusted models. We controlled for some confounding covariates, which could potentially affect outcome (sleep quality). In the first adjusted model (model 1), the confounding effects of age, sex, education, and marital status were controlled. In model 2, additional adjustment was made for BMI, smoking, diabetes, hypertension, heart diseases, KT/V, and dialysis duration. We used SPSS software (version 16; SPSS Inc., Chicago, IL, USA) for all statistical analyses.

## RESULTS

A total of 114 peritoneal dialysis patients were included in the present study. The mean age was 56.90 (standard deviation: 15.74) and 65% of patients were male. Our results indicated that 65% of peritoneal dialysis patients were poor sleepers. The main characteristics of patients in relation to sleep quality are presented in Table 1. There was no statistically significant difference between peritoneal dialysis patients with good sleep quality compared to those with poor sleep quality in terms of clinical and socio-demographic characteristics (*p*>0.05). As shown in Table 2, the dietary intake of nutrients was not significantly different between good and poor sleepers (*p*>0.05). However, the frequency of good and poor sleepers was significantly different across the level of macronutrients’ intake (*p*<0.05) (Table 3).

**Table 1:** Basic demographic and clinical characteristics of patients with normal and poor sleep quality.

Variables	Sleep quality		p-value*
	Good (N=40)	Poor (N=74)	
**Age (years)**	55.57±16.60	58.24±14.88	0.38
**Sex**			
Male	62.5	62.2	0.57
Female	37.5	37.8	
**Body mass index (kg/m^2^)**	25.33±5.60	25.26±3.97	0.93
**Educational status**			
0 - 5 years	45	48.7	0.89
5 - 12 ears	47.5	40.4	
>12 years	7.5	10.9	
**Marital status**			
Married	75	81.1	0.30
Single	25	18.9	0.30
**Smoking (yes)**	35	28.4	
**Comorbidities**			
Diabetes	35	48.6	0.11
Hypertension	57.5	78.4	0.01
Others	45	41.9	0.45
**Dialysis duration (months)**	45.07±32.68	36.89±25.06	0.14
**KT/V**	1.83±0.46	2.00±0.52	0.13

Notes: Values are presented as mean±SD and percentage for continuous and categorical variables;

* Resulted from chi-square for categorical and independent samples t-test or Mann-Whitney for continuous and non-normally distributed variables.

**Table 2:** Mean of nutrients’ intake in patients with good and poor sleep quality.

Variables	Sleep quality		p-value*
	Good (N=40)	Poor (N=74)	
Calorie	1811.89±713.61	2020.76±769.97	0.16
Carbohydrate	222.03±96.86	257.43±97.24	0.07
Protein	65.65±30.61	68.94±19.90	0.50
Fat	76.43±43.61	83.64±42.84	0.40
Cholesterol	266.52±170.55	287.92±218.03	0.60
Saturated fatty acid	23.10±14.87	26.43±23.32	0.42
Mono-unsaturated fatty acid	28.39±16.89	30.23±14.75	0.55
Poly-unsaturated fatty acid	17.45±12.60	17.85±9.34	0.85
Fiber	24.25±11.34	28.54±13.37	0.09
Vitamin A	617.31±929.81	554.10±492.17	0.64
Vitamin D	1.34±1.25	1.14±0.89	0.33
Vitamin K	208.84±216.25	237.48±369.24	0.66
Vitamin E	19.83±17.49	17.36±9.59	0.34
Vitamin C	84.36±58.30	101.47±63.72	0.17
Thiamin	1.38±0.71	1.50±0.50	0.27
Riboflavin	1.67±0.99	1.76±0.65	0.59
Niacin	13.96±6.61	15.26±4.96	0.25
Pantothenic acid	4.54±2.164	4.97±1.64	0.25
Vitamin B6	1.44±0.70	1.57±0.48	0.25
Biotin	23.23±13.41	24.94±9.81	0.45
Folic acid	454.30±211.51	470.41±159.70	0.65
Vitamin B12	4.08±2.81	4.26±2.48	0.72

Notes: Data are presented as mean±SD;

* Resulted from ANCOVA and p-value<0.05 has been considered significant, adjustment was made for age, gender, and calorie intake for all nutrients except for calorie (adjustment was made for age and gender).

**Table 3:** Sleep quality across the levels of macronutrients’ intake.

		Sleep quality		p-value*
		Good	Poor	
Protein	<median	42.6	57.4	0.10
	>median	29.1	70.9	
Carbohydrate	<median	48.1	51.9	0.008
	>median	23.6	76.4	
Fat	<median	48.1	51.9	0.008
	>median	23.6	76.4	
Fiber	<median	46.3	53.7	0.02
	>median	25.5	74.5	

Note:

* Resulted from chi-squared test.

According to logistic regression, higher level of dietary consumption of fiber (OR:2.53; 95% CI: 1.12-5.67; *p*<0.05), fat (OR:3; 95% CI:1.32-6.81; *p*<0.05), and carbohydrate (OR:3; 95% CI:1.32-6.81; *p*<0.05) was significantly associated with higher risk of poor sleep quality in crude model. In the fully adjusted model, the association between the intake of fiber (OR:2.95; 95% CI: 1.13-7.64; *p*<0.05), fat (OR:4.89; 95% CI: 1.70-14.09; *p*<0.05), and carbohydrate (OR:4.01; 95% CI: 1.52-10.55; *p*<0.05) with poor sleep quality remained significant (Table 4). However, there was no statistically significant association between protein intake (OR: 1.81; 95% CI: 0.82-4.00; *p*>0.05) and sleep quality, even after adjustment for confounders (*p*>0.05).

**Table 4:** Crude and multivariable adjusted odds ratios (95% confidence interval for ORs) of the association between macronutrients’ intake and sleep quality.

	Crude model		Model 1		Model 2	
	OR	95% CI	OR	95% CI	OR	95% CI
Protein	1.81	0.82-4.00	1.92	0.83-4.32	1.37	0.43-4.35
Carbohydrate	3*	1.32-6.81	3.68*	1.53-8.87	4.01*	1.52-10.55
Fat	3*	1.32-6.81	3.83*	1.51-9.70	4.89*	1.70-14.09
Fiber	2.53*	1.12-5.67	3.04*	1.29-7.17	2.95*	1.13-7.64

Notes: Model 1 - adjusted for socio-demographic variable (sex, age, marital status, educational status); Model 2 - further adjusted for lifestyle and clinical variables (comorbidities, energy consumption, dialysis duration, KT/V, cigarette smoking, BMI); Multivariate logistic regression model was used to estimate odds ratios with 95% confidence intervals for the association of the outcome and macronutrients’ intake;

* Represents significant association between nutrients’ intake and sleep quality.

## DISCUSSION

Sleep complications are frequent in ESRD patients undergoing dialysis. The results of the current study demonstrated a high prevalence of poor sleep quality in peritoneal dialysis. It was in direction with the findings of other studies^13-15^. A cross-sectional study by Allemand et al. (ANO)^13^ showed that 58.6% of elderly patients undergoing hemodialysis were poor sleepers. Another cross-sectional study among dialysis patients in Iran also indicated a high prevalence of poor sleep quality in peritoneal dialysis patients (86.6%)^14^. Previous studies have shown that poor sleep quality is associated with the risk of nutrition-related disorders such as obesity, cardiovascular disorders, and diabetes^16-19^. As a result, there has been a growing interest to explore the association between sleep quality and dietary intake. Interestingly, previous studies have shown that there is a reciprocal association between various aspects of sleep and dietary choices. For example, short sleepers are more likely to choose calorie-dense foods such as fats and refined carbohydrates^20,21^. Furthermore, it has been shown that unhealthy dietary habits like low intake of vegeTables and fish and high intake of confectionary are associated with poor sleep quality^22^. However, a balanced diet comprising fresh fruits, vegeTables, whole grains and low-fat protein sources can increase sleep quality^23,24^.

In spite of the high prevalence of sleep disorders in ESRD patients, a few number of studies have examined the association between diet and sleep quality, assessed with PSQI, in peritoneal dialysis patients. In this study, we reported a positive significant association between dietary intake of carbohydrate, fat and fiber with poor sleep quality, suggesting that patients who consume higher amounts of these nutrients are more likely to have poor sleep quality. Although, no association was found between dietary intake of protein and sleep quality. As far as we know, this is the first study that investigated the association between nutrients’ intake and sleep quality in peritoneal dialysis patients.

Some few studies in the general population have been investigated the association between nutrients’ intake and sleep; although, their results are controversial. A large cross-sectional study on middle-aged Japanese non-shift workers showed that low carbohydrate and high protein intakes were associated with difficulty maintaining sleep. According to the result of the study, low protein intake was associated with difficulty initiating sleep and poor sleep quality^25^. It has also been suggested that sleep duration is affected by relative intake of carbohydrates and proteins. Higher carbohydrate intake has been shown to be associated with long sleep duration; while, an inverse association has been observed for protein intake^26^. However, the results of a large-cross-sectional study on Chinese population showed a significant association between low carbohydrate and high fat intake with decreased sleep duration^27^. In contrast to the above-mentioned studies, no association was found between sleep quality and macronutrients’ intake among Bavarian population^28^. The differences in the result of various studies may result from differences in study design, studied population and approaches used for estimating dietary intakes or sleep quality. We also assume that differences in lifestyle and dietary patterns of various populations maybe explain the controversial results of these studies. As a result, further observational studies are required to explore the association between daily intake of nutrients and sleep quality.

The study had some limitations. First of all, the study sample was relatively small. Moreover, we could not exclude all potential confounders comprising chemical variables, physical, and psychological complications. We only focused on sleep quality; however, there are various aspects of sleep such as polysomnographic parameters. As a result, further large-scale studies are needed to confirm the findings in dialysis patients. In summary, the findings of the current study supported the hypothesis that nutrients’ intake affect sleep quality in dialysis patients; while, the association between dietary intake of various nutrients and sleep quality in the population was different from those have been observed in the general population. Our survey showed that there is a positive significant association between dietary intake of fiber, carbohydrate, and fat with poor sleep quality. However, our study failed to show any significant association between dietary protein intake and sleep quality.
